# A Simple Stochastic Model with Environmental Transmission Explains Multi-Year Periodicity in Outbreaks of Avian Flu

**DOI:** 10.1371/journal.pone.0028873

**Published:** 2012-02-17

**Authors:** Rong-Hua Wang, Zhen Jin, Quan-Xing Liu, Johan van de Koppel, David Alonso

**Affiliations:** 1 Department of Mathematics, North University of China, Taiyuan, Shan'xi, People's Republic of China; 2 Spatial Ecology Department, the Netherlands Institute of Ecology, Yerseke, The Netherlands; 3 Community and Conservation Ecology Group, University of Groningen, Groningen, The Netherlands; 4 Center for Advanced Studies, Spanish Council for Scientific Research, Blanes, Catalunya, Spain; Leeds University, United Kingdom

## Abstract

Avian influenza virus reveals persistent and recurrent outbreaks in North American wild waterfowl, and exhibits major outbreaks at 2–8 years intervals in duck populations. The standard susceptible-infected- recovered (SIR) framework, which includes seasonal migration and reproduction, but lacks environmental transmission, is unable to reproduce the multi-periodic patterns of avian influenza epidemics. In this paper, we argue that a fully stochastic theory based on environmental transmission provides a simple, plausible explanation for the phenomenon of multi-year periodic outbreaks of avian flu. Our theory predicts complex fluctuations with a dominant period of 2 to 8 years which essentially depends on the intensity of environmental transmission. A wavelet analysis of the observed data supports this prediction. Furthermore, using master equations and van Kampen system-size expansion techniques, we provide an analytical expression for the spectrum of stochastic fluctuations, revealing how the outbreak period varies with the environmental transmission.

## Introduction

Understanding the dynamics of infectious diseases in humans has become a increasing focus in public health science [1]–[3]. Despite a massive body of research on the epidemiology of seasonal influenza, overall patterns of outbreak and infection have not been fully understood, in particularly with regard to its multi-year periodicity. Disease outbreak, persistence, fadeout and transmission among species remain difficult to assess, because they not only depend on a huge variety of biological factors, e.g. virulence, immunity [4], but also on some abiotic processes, such as the characteristics of natural environments [5], [6], transport and immigration [7], [8]. In spite of all these inherent complexities, simple mathematical models can provide some very useful information for many infectious diseases including measles, mumps and rubella. From early deterministic compartmental models to more recent spatially structured stochastic simulation models [9], [10], dynamic models have impacted both our understanding of epidemic spread and public health planning.

Multi-year periodicity in epidemics is widely observed in time series from many cities with greatly varying climatic and demographic conditions [5], [11]–[13]. As reported previously [14], [15], multi-year periodicity and irregular fluctuations were related to both seasonal forcing and entrainment in nonlinear oscillatory and chaotic models. Deterministic models are typically assumed to be reasonable approximations for infinitely large, homogeneous populations, and arise from the analysis of mean field stochastic models. However, when one considers finite populations, stochastic interactions even within a well-mixed system introduce new phenomena. For example, disease persistence is determined by chance events when the number of individuals carrying the disease is small, during the early phases of disease invasion, or when total susceptible population size is reduced due to vaccination and/or immunity. In this case, even if invasion is predicted to be successful in deterministic models, i.e., the basic reproductive number (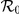) is larger than one, it may totally fail in the corresponding stochastic system, which means that observing a failed invasion in nature does not necessarily imply a population below the deterministic invasion threshold. In general, stochastic effects are quite prominent in finite populations, and remain important both in ecological [16]–[18] and epidemic dynamics [19]–[21]. Usually, individual-based and/or integer-based event-driven simulations [22] are conducted. However, simulations are inferior in several respects to careful mathematical analysis. For instance, a single simulation may not be representative of system average behavior but merely produce an outlier due to a rare combination of events [23]. Usually a huge ensemble of replicates are needed to obtain a good representation of the average behavior of the system. In fact, it is generally accepted that deeper insights are obtained from the mathematical analysis of stochastic systems.

Recently, a method, the so-called van Kampen's system-size expansion, which is based on a simple individual-based mathematical formulation of stochastic dynamics, has been applied to investigate stochastic population dynamics [21], [24]–[28]. This general mathematical framework provides an exact description of individual-based (integer-based) event-driven stochastic dynamics [22]. More recently, these methods have been applied to epidemiology, which has helped to understand the effects of stochastic amplification [21], [29] and seasonal forcing [30]–[32] on disease outbreaks. However, most of these studies are based on single species models, and mainly considered demographic stochasticity and seasonality. Roche *et al* [33], however, have shown that epidemic outbreaks and migrations are not synchronous, which points to the fact, that, in wild birds, virus persistence in the water should play a major role in the epidemiological cycles. This approach to characterize disease fluctuations provides a unique opportunity to investigate the effects of stochasticity imposed by finite population numbers on disease persistence and outbreaks both in single- and multi-species systems.

Here, we estimate the outbreaks period of avian influenza in North Americas with a wavelet method, which reveals 4–8 year periodicity from empirical data. To explain this, we first develop a fully stochastic two species' avian influenza model (host and virus) with two routes of transmission: environmental indirect transmission and direct transmission through contact between individuals within the wild bird population. Then, we provide a prediction for the dominant period of disease oscillations by analytically calculating the power spectral density from a stochastic Fokker-Planck equation. From a geographically (environmentally) and temporally restricted data, the model gives general insights into long-term patterns of disease dynamics in wild bird populations. Some conclusions may also apply to other infectious diseases characterized by two transmission routes. Our analysis sheds new light on the importance of environmental transmission for avian influenza outbreaks and persistence. Our results show that, in principle, it is possible to reduce the frequency and intensity of the outbreaks of avian influenza by controlling the environmental route of transmission.

## Methods

### The stochastic SIR model with environmental transmission

In avian influenza, susceptible hosts are not only infected by direct contact with infected individuals with avian influenza viruses (AIV), but also by virus particles that persist in the aquatic environment. AIV are transmitted via the fecaloid route of the host and subsequent drinking or filtering of water while feeding [3]–[36]. As a consequence, epidemic outbreaks are not necessarily induced by the arrival of infected hosts in the population, but can also result from virus particles that persist in the environment. The persistence and subsequent outbreak of viral particles in the aquatic environment is determined by several deterministic causes [34]. However, stochasticity should also play an important role [21], [34] because the processes controling the densities of viral particles, such as ingestion and shedding by hosts, and virus decay in the environment, are essentially probability processes. Accordingly, we consider the density of virus particles in the environment as a separate stochastic variable, which we couple to the dynamics of host infection through the environmental transmission rate. We provide a detailed description about the way in which, as well as the assumptions under which this is done in section A3 of Methods S1.

To better understand the effects of demographic stochasticity and virus persistence in the environment on epidemic outbreaks and extinction, we describe virus population dynamics and environmental transmission using an explicit stochastic host-pathogen model that assumes global mixing, i.e, random contact between individuals. Hence the epidemiological dynamics of the host falls within the susceptible-infected-recovered (SIR) framework [37], in which each individual is either susceptible (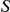), infectious (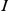) or recovered (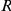), but infection occurs through two different routes: direct contact between susceptible (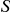) and infected (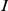) individuals, and external infection from the environment (as found the case in AIV in ducks or other waterfowl). For the former, the rate of infection can be expressed by 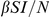, where 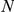 is the size of the host population and 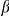 is the transmission rate. For the latter, according to the Poison distribution, the transmission rate per susceptible individual should be given by 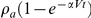 (see section A3 of Methods S1, and also the discussion in Ref. [34] for details), which is an increasing function of the virus number 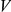 per unit volume. In fact, it can be simplified into its leading-order term using a Taylor expansion, which leads to 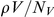 (here 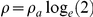, see Eq. (A29) in section A3 of Methods S1), where 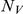 is a typical reference virion concentration in the water (see section A3, Methods S1), and 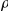 is the environmental transmission rate (see Methods S1, for details). For most host-parasite systems, environmental transmission is often represented as a frequency-dependent process, which means that the transmission rate depends on the frequency of infected vectors in the environment rather than on its absolute number or concentration as is the case for density-dependent transmission [38]. Similar transmission rates have been considered in malaria [39], dengue fever [35], West Nile Virus [36], and avian influenza [34].

Although births and deaths are intrinsically distinct events, we assume, for simplicity, that host birth and death rates have the same value 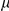, which means that the total population size 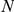 is kept constant. In sum, the dynamics of the disease in the host population can be expressed by the following elemental events:
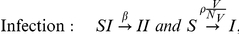


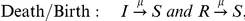


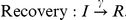



where 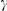 is the recovery rate. Since host population (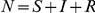) is kept constant, after any individuals dies, at the same time, a new susceptible host will be born in order to keep the total population 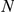 constant. Therefore, since 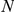 can be just seen as a model parameter, we eliminate the variable recovered individual 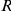 by using 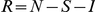 from our equations.

Our individual-based stochastic model fully integrates the abundance of virus particles in the environment into the SIR framework. Virus particles 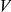 are shed by infected ducks (shedding rate is 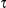), then virion concentration decays in the environment at rate 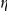. To keep the model general and applicable to other types of pathogens, and to consider, effectively, the possibility of replication of the virus in alternative hosts whose concentrations are not explicitly modeled, we introduce a production rate, 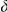. In this context, this parameter takes into account the ability of the virus to replicate outside of the specific host that is consider in the model. It is important to remark that in the limit of 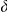 vanishing small, all our conclusions still hold (see section A3 of Methods S1). Therefore, its dynamics can be captured by:
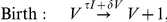


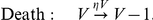



**Figure 1:**
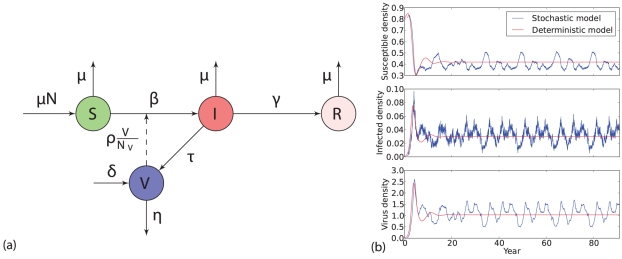
Stochastic SIR-V model. (a) Schematic diagram of the baseline SIR host-parasite model with direct and environmental transmission. The symbol 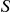 represents the susceptibles, 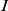 and 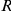 represent the infected and recovered individuals, respectively, and 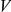 is the virus concentration in the environment. For host, there is equal birth rate and death rate 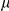. (b) A realization of a stochastic SIR-V model and its deterministic counterpart. The parameters used in the simulations are 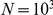, 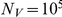, 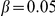, 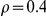, 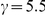, 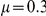, 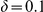, 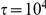 and 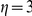. Disease parameters correspond to avian influenza epidemics derived for typical water-borne transmission from [33] and [2]. Detailed descriptions of model parameters and sources for their numerical values are presented in tab:2. The deterministic curve was generated by integrating the mean field equations (5), and stochastic simulation was implemented with Gillespie algorithm [22] with rates listed in Table 1.

All the transitions of the host and the virus associated with their corresponding rates are illustrated graphically in Fig. 1(a).

The basic ingredients of our new framework are the susceptible 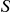, infected 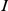 and the virus 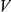 whose actual numbers are respectively denoted as 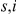 and 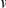, which are all of them integer. The general state of the system is then denoted as 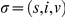. All of the processes taking place in this model and their corresponding rates are summarized in Table 1.

**Table 1:** List of events associated with transition rates.

Event	Transition	Rate	Probability in 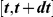
Direct infection	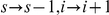	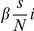	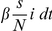
Environment infection^a^	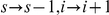	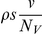	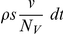
Death of recovered	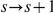	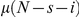	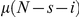 dt
Death of infected^b^	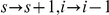	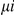	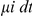
Recovery	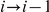	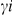	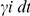
Birth of virus	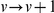	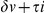	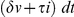
Death of virus	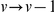	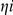	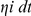

a Note that here we consider it as a frequency dependent.

b There is no empty site, and the population size 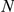 is constant, thus a new susceptible individual will be born once an infective individual dies.

The transition probability per unit time from state 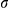 to the state 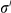 will be denoted as 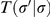, in which 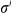 is obtained by shifting each state variable in 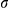 by +1 or −1. According to the information of Table 1, the events occurring in the system can be divided into three groups:

1. Infection

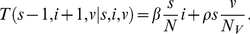
(1)

1. Death/Birth

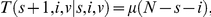


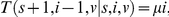


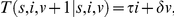


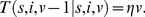
(2)

1. Recovery

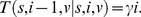
(3)Having defined the transition rates between different states by Eq. (1)–(3), now we can construct a master equation describing the temporal evolution of the system. It takes the general form [16], [17], [21], [25]–[28]

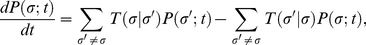
(4)


where 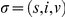 represents the state of the system, 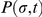 is the probability of the system in the state 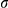 at time 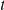, and the change of this quantity with time is given by a balance between the sum of transitions into the state 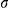 from all the other states 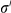, and minus the sum of transitions out of the state 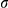 into all the other states 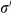.

So far we have formulated a fully stochastic host-parasite model with both direct and indirect environmental transmission, assuming well-mixed conditions. Given the specified analytical formulations for transition probabilities 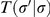, the master equation (4) accurately describes the temporal evolution of the probability 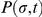. This model can now be investigated by a combination of simulation, by using Gillespie algorithm [22], and, analytically, by performing the van Kampen's system-size expansion [21], [25] of the master equation. Both methods allow quantitative prediction of the power spectrum of the time fluctuations of each of the system variables, and, therefore, of the dominant period of recurrent epidemic outbreaks.

Using van Kampen's system-size expansion of the stochastic dynamics, as discussed in section A1 of Methods S1, we can derive the deterministic equations. The stability of the steady states of this system is tractable, and can be obtained by deriving the deterministic limit (see subsection A1.1 of Methods S1). The next-to-leading order gives the linear stochastic differential equations–Fokker-Planck equation, which can be analyzed using the Fourier method. Now we start by introducing the new variables:
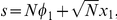


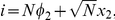


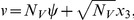



where 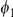, 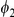, 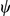 are the fractions of the susceptible hosts, the infected hosts and viruses in the environment, respectively, with 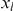 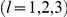 describing the stochastic corrections to the variables 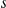, 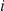 and 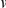. Full technical details of model analysis are given in the section A1 of Methods S1. To leading order, the deterministic equations for the fractions are
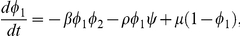


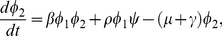
(5)

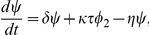



where 
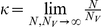
.

It is simple to verify that these equations have a trivial fixed point 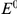:
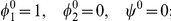



and a unique non-trivial fixed point 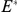:
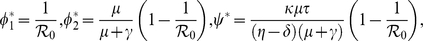



where 
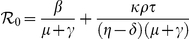
 is the basic reproductive number. From the stability's analysis in section A2 of Methods S1, we know when 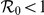, the trivial fixed point 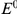 is stable; when 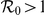, the non-trivial fixed point 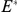 exists and is stable.

### The periodicity of the stochastic model

It is important to investigate whether the existence of a stable fixed point in the deterministic system generates oscillations and multi-year periodicity in the corresponding stochastic system. In order to investigate this and describe the stochastic fluctuations of the system by an analytical method, the higher-order terms should be included in the van Kampen system-size expansion [25]. As shown in the section A1 of Methods S1, the fluctuations obey a linear Fokker-Planck equation, which is equivalent to a set of Langevin equations having the form
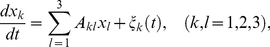
(6)


where 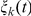 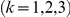 are Gaussian white noises with zero mean. In the same way, the cross-correlation structure is determined by the expansion, which satisfies 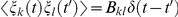. As mentioned above, we are interested in evaluating these fluctuations at the non-trivial fixed point of the deterministic system. For that reason, we evaluated the entries of the Jacobian matrix 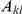 and 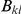 of the noise covariance matrix at this stable fixed point. Explicit expressions for these two matrices are given in the supporting information in subsection A1.2 of Methods S1.

The Langevin equations (6) describe the temporal evolution of the normalized fluctuations of variables around the equilibrium state. By Fourier transformation of these equations, we are able to analytically calculate the power spectral densities (PSD) that correspond to the normalized fluctuations, independent of community size 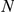. By taking the Fourier transform of Eqs. (6), we transform them into a linear system of algebraic equations, which can be solved, after taking averages, into the three expected power spectra of the fluctuations of the susceptible, infectious and viral densities around the deterministic stationary values:
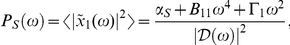


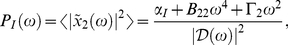
(7)

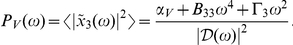



The complete derivation of these PSDs and detailed descriptions about the way the functions 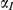, 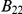, 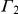, and 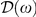 depend on model parameters are discussed in section A1.3 of Methods S1.

### Wavelet power spectrum

Unlike Fourier analysis, wavelet analysis is well suited for the study of signals whose spectra change with time. This time–frequency analysis provides information on the different frequencies (i.e. the periodic components) as time progresses [40], [41]. The wavelet power spectrum estimates the distribution of variance between frequency, 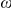, and different times, 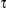.

If we denote the time-series as 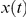, then the wavelet transform of a signal 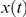 is defined as:
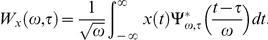



In the definition, parameters 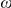 and 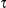 denote the dilation (periodicity) and translation (time shift position). 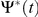 denotes the wavelet functions. There are three wavelet basis functions (Morlet, Paul and DOG) commonly used in the wavelet analysis. The Morlet wavelet is the one used in our analysis. Cazelles *et al* [40] presents a detailed description of the wavelet power spectrum method and a summary of its applications to disease and ecological data.

## Results

### Prevalence of influenza A viruses in wild ducks over time

**Figure 2:**
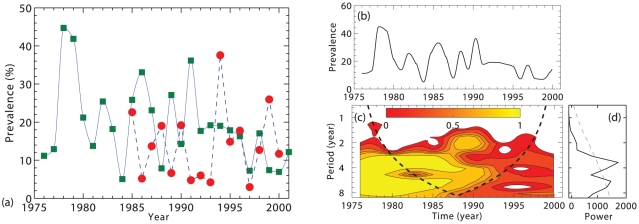
Temporal periodicity analysis of avian influenza in North America using the wavelet method. (a) Yearly prevalence of influenza A virus for wild ducks from 1976 to 2001 and for shorebirds from 1985 to 2000, where the data with green square and red circle symbols represent wild duck and shorebird, respectively. Annual prevalence was calculated as a percentage of the total number of samples tested for a given year that contained influenza A virus. We have redrawn this figure here with data kindly provided by Dr. Webster [13]. Panel (b) shows the time series with yearly prevalence of influenza A virus in wild ducks from 1976 to 2001. (c) The wavelet spectrum analysis corresponds to time series of panel (b), where time runs along the 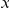-axis and the contours limit areas of power at the periods indicated in the 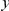-axis. High power values are colored in dark red; yellow and green denote intermediate power; cyan and blue, low. Note the bold continuous black line is known as the cone of influence and delimits the region not influenced by edge effects. Only patterns within these lines are therefore considered reliable. Finally, the right panel (d) corresponds to the average wavelet spectrum (black line; see section: wavelet power spectrum) with its significant threshold value of 5% (dotted line). Wavelet software provided by C. Torrence and G. Compo, is available at http://www.paos.colorado.edu/research/wavelets/.

Previous studies over 15 years from 1976 to 1990 established a cyclic pattern of occurrence of influenza A viruses in wild ducks [42], with high prevalence in some years followed by reduced prevalence in subsequent years. Avian influenza data of a yearly time series is described in Ref. [13], for wild aquatic birds from 1976 to 2001 in North America. Those records contain samples collected on wild ducks and shorebirds. To determine whether these patterns show multi-year periodicity, we examined avian influenza prevalence over the period from 1976 to 2001, as is shown in Fig. 2.

**Figure 3:**
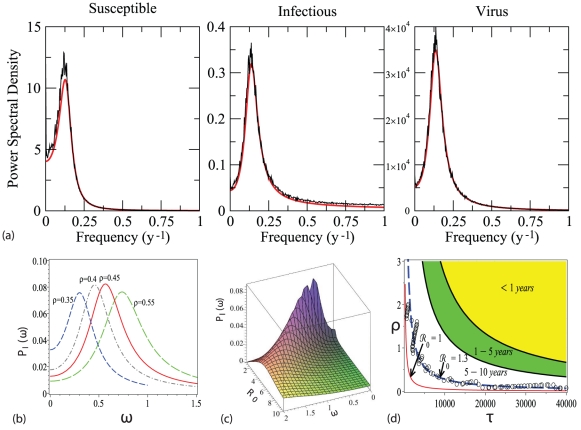
Power Spectral Density (PSD). (a) Comparisons between the theoretical prediction of the PSD (Eq. (7)) and the average PSD calculated from full stochastic simulation as the one shown in Fig. 1(b), for the fluctuations of the total number of the susceptible, the infected and the virus. The black lines represent the power spectra of time series obtained from stochastic simulations, and red lines represent the analytical prediction. The parameters are listed in Table 2 and 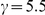, where 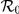 is equal to 2.387, and a main oscillatory period about 7 years. (b) Changes in the PSD as a function of an increasing environmental transmission rate with 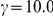. (c) Three-dimensional representation of the PSD for the variable 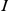, (Eq. (7)), for a continuum of values of 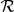 on the 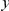 axis, with the restriction 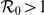. (d) Dominant period and persistence of the disease as a function of parameters 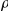 and 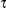. Here we divide the domain of the parameter space where sthochastic fluctuations occur in three different regions characterized by periods less than 1, from 1 to 5, from 5 to 10 years, respectively. We also represent the hyperbolic-shaped instability boundary, separating the domain of disease persistence (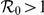) from the region of disease extinction (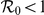), which is determined by the basic reproductive number 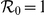 in the deterministic system (5). The same boundary can be calculated through simulation for the full stochastic model. It corresponds approximately to 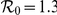. Symbols (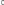) represent 100-year-long simulations, where the transient dynamics have been discarded (the first 50 years).

The data on aquatic wild birds revealed a clear periodicity in the outbreaks of avian influenza in agreement with literature [13]. These periodic patterns are confirmed from the case records through wavelet analysis (see Fig. 2(b)), as well as through its wavelet power spectrum analysis versus the frequency with the largest long-term detectable power (Fig. 2(c)and (d) ). Wavelet analysis performs a time-scale decomposition of a time signal, which involves the estimation of the spectral characteristics of the signal as a function of time. It reveals how the different periodic components of the time series change over time. The oscillations of avian influenza A virus in ducks species have a considerable variation as periodicity during these years. However, the wavelet analysis based on these data reveals significant multi-annual cycles from 2 to 8 years. By using our model predictions with reasonable parameter values (presented in Table 2), we can estimate the environmental transmission rate, 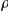, that yields fluctuating periods ranging from 2 to 8 years (see curves in Fig. 3(c and d) for different values of 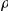). For instance, it should be lower than 0.42 year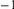 for a reproductive number equal to 2.4, 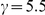, and the rest of parameters chosen according to Table 2.

**Table 2:** The definitions of the parameters in this model and their values for the special case (AIV).

Symbol	Definition	Value/range	Unit	Source a
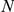	host population size	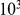	duck	
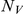	viral reference concentration	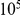	virion ml 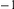	
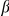	direct transmissibility	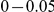	duck 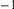 year 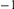	[33]
				[2]
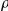 b	environmental transmissibility	0 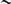 0.425	year 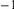	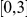 (years 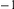 )
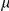	host birth and death rate	0.3	year 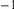	[55]
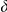	virus replication rate	0.1	year 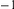	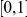 (years 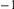 )
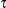	virus shedding rate	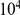	virion/duck/day	[56], [57]
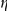	virus clearance rate	3	year 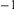	[58]
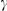	recovery rate	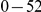	year 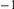	[34]

a Parameter values are based on empirical studies in literature. Since no data are available for 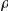 and 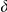, we let them vary within a reasonable range. We have studied how their values influence the patterns of interest (see Fig. 3).

b See Methods S1, section A3 for its biological significance.

### Effect of stochasticity and environmental transmission on disease outbreaks

Direct comparison of the deterministic and stochastic simulations reveals that demographic noise and environmental transmission can induce rich multi-period patterns, corresponding to deterministically damped oscillations (see Fig. 1(b)). Our analysis can help us to understand the effect of indirect transmission on the type of expected fluctuations of disease incidence. We compared the analytical predictions for the PSDs to simulated results in Fig. 3(a), using biologically reasonable parameter values (see Table 2). Our results reveal very good agreement between predictions and stochastic simulations.

The original PSD formula (7) further allows us to examine how the period of the epidemic outbreak varies with changes of the environmental transmission rate 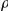. We show in Fig. 3(b) that, for typical parameters of avian influenza, as listed in Table 2, increased environmental transmission rate 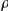 can enhance the frequency of disease outbreaks. We can see from Fig. 3(c,d) that, within the deterministic model, the effects of the basic reproductive number on outbreak periodicity of the disease are most pronounced when the pathogen invasion is close to the critical value (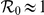). Furthermore, the PSD surface becomes flatter as the basic reproductive number 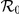 increases, indicating that more frequencies are involved in the stochastic fluctuations, and that the overall variance of infected time series is more evenly distributed among these frequencies. Simultaneously, as the basic reproductive number increases, the dominant period decreases (the dominant frequency increases), as is elucidated in Fig. 3(c an d). Finally, coherence disappears and the PSD becomes totally flat at larger values of the basic reproductive number, 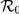. In that regime, time fluctuations around average stationary values do not show a dominant frequency and become white noise.

We characterize the region of the parameter space that allows for disease persistence both in the deterministic model (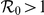) and, through simulations, in the corresponding stochastic system. We also map the dominant period, which is calculated with the inverse of the frequency at which the PSD peaks (the dominant frequency) in year units (see Fig. 3(d)). From Fig. 3(d), one can see that the larger the basic reproductive number of the deterministic model is, the higher outbreak frequencies in the stochastic model tend to be. This can also be seen by looking at the analytical prediction of the PSD from Eq. (7) (see Fig. 3(c)). Furthermore, we notice that disease stochastic extinction occurs even if the basic reproductive number is slightly above its critical deterministic threshold. This is a common difference between deterministic models and its finite-size stochastic counterparts which is usually difficult to quantify. Through simulation, we have have approximated the boundary separating disease persistence from stochastic extinction by the curve of 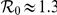 (see Fig. 3(d)).

These reported results are robust to changes in model parameters within the ranges given in Table 1. For instance, when we take 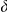 to zero, the parameter representing pathogen self-maintenance in the environment, very minor changes are seen in the predicted power spectra. For details on model sensitivity to parameter changes, see section A4 of Methods S1.

## Discussion

In this paper, we have developed a general, fully stochastic host-pathogen model with two routes of transmission: individual-to-individual and environmental transmission. Our theory provides a simple, plausible explanation for the phenomenon of multi-year periodic outbreaks of avian flu. Even in the absence of external seasonal forcing, our theory predicts complex fluctuations with a dominant period of 2 to 8 years for reasonable parameters values, which essentially depends on the intensity of environmental transmission. Since our model does not consider the specificities of bird migration or seasonal reproduction in any way, in fact, it applies to any infectious disease with two routes of transmission, such as cholera. This further justifies the analysis we have done which assures that infectious agents in the environment can not only persist in the environment but also reproduce.

Practically all infectious diseases exhibit fluctuations. Childhood diseases [9], [20], dengue fever [43], cholera [44], [45], malaria [39], [46], and avian influenza [34] are but a few examples where disease incidence strongly fluctuates. Emerging largely from a deterministic framework, the standard paradigm is that seasonal and/or climatic extrinsic forcing and intrinsic host-pathogen dynamics are both required to understand the character of different types of disease oscillations from regular to rather erratic patterns [15]. However, more recently, it has become clear that the interaction between the deterministic dynamics and demographic stochasticity is fundamental to understand realistic patterns of disease [20] including vaccine-induced regime shifts [21].

Breban *et al* [34] developed a host-pathogen model for avian influenza combining within-season transmission dynamics with a between-season component that describes seasonal bird migration, and pulse reproduction. In their model, virus dynamics in the environment is modeled as a deterministic process. Their model is designed to apply specifically to avian flu. By contrast, our model applies more generally, and considers a much simpler dynamics (without either seasonal pulse reproduction or seasonal bird migration). In spite of these simplifications, we are still able to reproduce with similar accuracy realistic patterns of disease fluctuations for avian flu. Although our explanation is simpler, both models show that the interplay between the stochastic component of disease dynamics and environmental transmission is essential to understand the erratic outbreak patterns of avian influenza, characterized by dominant periods from 2 to 8 years (see Fig.2c). Here we confirm this previous conclusion [34], and show that it does not critically depend on bird migration and pulse reproduction. In addition, in this paper, we are able to predict analytically how the whole spectrum of such fluctuations depends on model parameters.

In particular, in order to derive the power spectrum, we have applied van Kampen expansion [25] to the full stochastic model. This method allows to study the correct interaction between the deterministic and the stochastic components of the system in a formal way in the case of finite populations. We have shown that the predicted power spectrum is in excellent agreement with model simulations for realistic parameter values. In particular, our study reveals that higher values of environmental transmission increase the frequency of epidemic outbreaks.

Our general framework can be seen essentially as a stochastic SIR model with two types of disease transmission: individual-to-individual and environmental transmission, which takes into account the fact that disease agents are released to the environment by infected individuals and, once there, they follow a simple dynamics of decay and self-maintenance. Of course, virus particles cannot self-reproduce independently from the host. In our application to avian influenza, this term would take into account virus reproduction in other host species different from the focal host. In our model, the influence of the “reproduction” parameter on our predicted power spectrum is very small (see Methods S1). In addition, our framework readily apply to the large number of infectious diseases where reproduction of the infectious agents in the environment is not negligible, and the interplay between these two routes of transmission is known to be important [47]–[53].

Our work points to the fact that seasonal forcing, taking into account pulse reproduction and seasonal bird migration, is not essential to understand avian flu fluctuating patterns of disease incidence. We argue that this is basically a consequence of the inherent stochasticity of the system. This type of ‘endogenous’ stochastic resonance [28] has been also described in childhood diseases [21]. This result does not mean that seasonal dynamics is not important in realistic situations. Migration and seasonal reproduction are the most reasonable minimal ingredients of any disease model with applications to migratory birds, and they surely control other important processes in these systems.

The extend to which the seasonal cycle controls disease fluctuating patterns has been recently studied in a fully stochastic framework (both SIR [31] and SEIR [32]) with applications to childhood diseases. These powerful analytic methods apply also to infectious diseases with two transmission routes, such as avian influnza, and further work on this area should be done. However, these preliminary studies have already revealed that a complex interaction of seasonal forcing and the inherently stochastic, non-linear dynamics of the disease occurs only in very restricted areas of the parameter space, in particular, close to bifurcation points [31]. For the most part of the parameter space, apart from a rather thin seasonal peak, the predicted, non-forced power spectral density (PSD) agrees reasonably well with the PSD averaged over seasonally forced, stochastic model simulations [21], [31], [32].

Simple non-linear systems have the potential to predict the complex spatio-temporal patterns observed in nature. The role of stochasticity and the way it interacts with nonlinearity are central issues in our attempt to understand such complex population patterns. As new tools and approaches become available [21], [23], [31], [32], [54], here we have argued that the interaction of external forcing with nonlinearity should be addressed within a fully stochastic framework. Going back to avian influenza, we may well be in a situation where seasonal migration and reproduction are rather punctual events that would probably lock the phase of disease fluctuations without strongly influencing the way the overall spectral power is distributed among the different frequencies at play, which is basically determined by the intrinsic non-linear stochastic dynamics of the system. This hypothesis applies to other infectious diseases as well as to, quite generally, fluctuating populations in ecological systems. It deserves, on itself, further investigation.

## Supporting Information

Methods S1 **In the supporting information file, we provide, essentially, detailed mathematical derivations of the different theoretical results presented in the main text.** Supporting information is divided in four sections. The first one is devoted to the link between the deterministic and stochastic description of the system and the system-size expansion used to calculate power spectral densities. The second one analyzes the dynamical stability of fixed points of the deterministic system. The third one justifies the functional form used to represent environmental transmission, and finally, the last one includes a sensitivity analysis of the main fluctuation periodicity with respect to two model parameters.
(PDF)
