# Supplementary material for: A Simple Stochastic Model with Environmental Transmission Explains Multi-Year Periodicity in Outbreaks of Avian Flu
Source: PLoS One. 2012 Feb 17;7(2):e28873. doi: 10.1371/journal.pone.0028873 (PMC3281819; doi:10.1371/journal.pone.0028873)
Supplement: Methods S1 — In the supporting information file, we provide, essentially, detailed mathematical derivations of the different theoretical results presented in the main text. Supporting information is divided in four sections. The first one is devoted to the link between the deterministic and stochastic description of the system and the system-size expansion used to calculate power spectral densities. The second one analyzes the dynamical stability of fixed points of the deterministic system. The third one justifies the functional form used to represent environmental transmission, and finally, the last one includes a sensitivity analysis of the main fluctuation periodicity with respect to two model parameters. (PDF) [file pone.0028873.s001.pdf]

A simple stochastic model with environmental transmission explains multi-year periodicity in outbreaks of avian flu

Rong-Hua Wang, Zhen Jin, Quan-Xing Liu,
Johan van de Koppel, David Alonso

Methods S1: Supporting Information

A1 Stochastic Methods

A1.1 The deterministic limit

Detailed extension about master equation (4) is

$$\begin{aligned}\frac{dP(s,i,v;t)}{dt} &= T(s,i,v|s+1,i-1,v)P(s+1,i-1,v;t)+T(s,i,v|s-1,i,v)P(s-1,i,v;t)\\ &+T(s,i,v|s-1,i+1,v)P(s-1,i+1,v;t)+T(s,i,v|s,i+1,v)P(s,i+1,v;t)\\ &+T(s,i,v|s,i,v-1)P(s,i,v-1;t)+T(s,i,v|s,i,v+1)P(s,i,v+1;t)\\ &-[T(s-1,i+1,v|s,i,v)+T(s+1,i,v|s,i,v)+T(s+1,i-1,v|s,i,v)\\ &+T(s,i-1,v|s,i,v)+T(s,i,v+1|s,i,v)+T(s,i,v-1|s,i,v)]P(s,i,v;t),\end{aligned} \quad \text{(A1)}$$

which gives a complete description of the time evolution of the non-spatial model, from which we can obtain a deterministic version of the model. A straightforward way is to multiply Eq. (A1) by s, i and v in turn, and subsequently to sum over all allowed values of s, i and v, and to take all the boundary values zero [26]. This gives equations for the mean values $S = \langle s\rangle$, $I = \langle i\rangle$ and $V = \langle v\rangle$. For $S = \langle s\rangle = \sum_{s,i=0}^{N}\sum_{v=0}^{N_v} sP(s,i,v;t)$, the mean-field theory takes the form

$$\begin{aligned}\frac{dS}{dt}=\frac{d\langle s\rangle}{dt} &= \sum_{s,i=0}^{N}\sum_{v=0}^{N_v}[T(s+1,i,v|s,i,v)+T(s+1,i-1,v|s,i,v)]P(s,i,v;t)\\ &-\sum_{s,i=0}^{N}\sum_{v=0}^{N_v}T(s-1,i+1,v|s,i,v)P(s,i,v;t),\end{aligned} \quad \text{(A2)}$$

A similar way gives the equations for $I = \langle i\rangle$ and $V = \langle v\rangle$:

$$\begin{aligned}\frac{dI}{dt} &= \sum_{s,i=0}^{N}\sum_{v=0}^{N_v}T(s-1,i+1,v|s,i,v)P(s,i,v;t)\\ &-\sum_{s,i=0}^{N}\sum_{v=0}^{N_v}[T(s+1,i-1,v|s,i,v)+T(s,i-1,v|s,i,v)]P(s,i,v;t),\end{aligned} \quad \text{(A3)}$$

and

$$\frac{dV}{dt}=\sum_{s,i=0}^{N}\sum_{v=0}^{N_v}T(s,i,v+1)P(s,i,v;t)-\sum_{s,i=0}^{N}\sum_{v=0}^{N_v}T(s,i,v-1)P(s,i,v;t). \quad \text{(A4)}$$

So far there is no approximation made in the derivation of Eqs. (A2)–(A4), however, we can now substitute those transition rates of Eqs. (1)–(3) into these equations and take the mean-field limit $N, N_V \to \infty$, which allows us to take the replacement $\langle si \rangle = \langle s \rangle \langle i \rangle$. After introduce the fractional variables

$$\phi_1 = \lim_{N\to\infty} \frac{S}{N}, \quad \phi_2 = \lim_{N\to\infty} \frac{I}{N}, \quad \psi = \lim_{N_V\to\infty} \frac{V}{N_V}, \tag{A5}$$

we can thus write down the deterministic equations as

$$\begin{aligned} \frac{d\phi_1}{dt} &= -\beta\phi_1\phi_2 - \rho\phi_1\psi + \mu(1-\phi_1), \\ \frac{d\phi_2}{dt} &= \beta\phi_1\phi_2 + \rho\phi_1\psi - (\mu+\gamma)\phi_2, \\ \frac{d\psi}{dt} &= \delta\psi + \kappa\tau\phi_2 - \eta\psi, \end{aligned} \tag{A6}$$

where let constant $\kappa = \lim_{N,N_V\to\infty} \frac{N}{N_V}$.

A1.2 The system-size expansion and analysis of the fluctuations

Now we are in a position to implement the application of the van Kampen [25] system-size expansion to study the fluctuations. First, we introduce new continuous variables x_1, x_2, x_3, in place of the previously used discrete variables s, i, v and make following replacement in the transition probabilities which appear in the master equation

$$\begin{aligned} s &= N\phi_1 + \sqrt{N}x_1, \\ i &= N\phi_2 + \sqrt{N}x_2, \\ v &= N_V\psi + \sqrt{N_V}x_3. \end{aligned}$$

Next, defining a new probability distribution function Π by $P(s,i,v;t) = \Pi(x_1,x_2,x_3;t)$, which implies that

$$\frac{dP}{dt} = \frac{\partial\Pi}{\partial t} - \sqrt{N}\frac{d\phi_1}{dt}\frac{\partial\Pi}{\partial x_1} - \sqrt{N}\frac{d\phi_2}{dt}\frac{\partial\Pi}{\partial x_2} - \sqrt{N_V}\frac{d\psi}{dt}\frac{\partial\Pi}{\partial x_3}. \tag{A7}$$

When using this formalism it is useful to rewrite the master equation (A1) in another way that is by introducing the step operators [21, 26]

$$\begin{aligned} \mathcal{E}_s^{\pm1} f(s,i,v) &= f(s\pm1,i,v), \\ \mathcal{E}_i^{\pm1} f(s,i,v) &= f(s,i\pm1,v), \\ \mathcal{E}_v^{\pm1} f(s,i,v) &= f(s,i,v\pm1), \end{aligned}$$

the Eq. (A1) with transition rates of Eq. (1)–(3) can be rewritten as

$$\begin{aligned}\frac{dP(s,i,v;t)}{dt} &= [(\mathcal{E}_s\mathcal{E}_i^{-1}-1)T(s-1,i+1,v|s,i,v)+(\mathcal{E}_s^{-1}-1)T(s+1,i,v|s,i,v)\\&+(\mathcal{E}_s^{-1}\mathcal{E}_i-1)T(s+1,i-1,v|s,i,v)+(\mathcal{E}_i-1)T(s,i-1,v|s,i,v)\\&+(\mathcal{E}_v^{-1}-1)T(s,i,v+1|s,i,v)+(\mathcal{E}_v-1)T(s,i,v-1|s,i,v)]P(s,i,v;t)\\&= \{(\mathcal{E}_s\mathcal{E}_i^{-1}-1)[\beta\frac{s}{N}i+\rho s\frac{v}{N_V}]+(\mathcal{E}_s^{-1}-1)\mu(N-s-i)+(\mathcal{E}_s^{-1}\mathcal{E}_i\\&-1)\mu i+(\mathcal{E}_i-1)\gamma i+(\mathcal{E}_v^{-1}-1)(\tau i+\delta v)+(\mathcal{E}_v-1)\eta v\}P(s,i,v;t). \qquad \text{(A8)}\end{aligned}$$

Expanding the step operators $\mathcal{E}_s^{\pm1}$ and $\mathcal{E}_i^{\pm1}$ in a power series in $N^{-1/2}$, $\mathcal{E}_v^{\pm1}$ in $N_V^{-1/2}$,respectively,

$$\begin{aligned}\mathcal{E}_s^{\pm1} &= 1\pm\frac{1}{\sqrt{N}}\frac{\partial}{\partial x_1}+\frac{1}{2N}\frac{\partial^2}{\partial x_1^2},\\\mathcal{E}_i^{\pm1} &= 1\pm\frac{1}{\sqrt{N}}\frac{\partial}{\partial x_2}+\frac{1}{2N}\frac{\partial^2}{\partial x_2^2},\\\mathcal{E}_v^{\pm1} &= 1\pm\frac{1}{\sqrt{N_V}}\frac{\partial}{\partial x_0}+\frac{1}{2N_V}\frac{\partial^2}{\partial x_3^2},\end{aligned}$$

and then substituting these expanding equations into Eq. (A8), drawing a comparison of which with Eq. (A7) order by order yields the so-called macroscopic equations

$$\frac{d\phi_1}{dt}=f_1(\phi_1,\phi_2,\psi),\quad \frac{d\phi_2}{dt}=f_2(\phi_1,\phi_2,\psi),\quad \frac{d\psi}{dt}=f_3(\phi_1,\phi_2,\psi), \qquad \text{(A9)}$$

to leading order, where

$$\begin{aligned}f_1(\phi_1,\phi_2,\psi) &= -\beta\phi_1\phi_2-\rho\phi_1\psi+\mu(1-\phi_1),\\f_2(\phi_1,\phi_2,\psi) &= \beta\phi_1\phi_2+\rho\phi_1\psi-(\mu+\gamma)\phi_2,\\f_3(\phi_1,\phi_2,\psi) &= \delta\psi+\kappa\tau\phi_2-\eta\psi, \qquad \text{(A10)}\end{aligned}$$

which are indeed the equations (A6). Therefore, an alternative and systematic way of obtaining the mean-field equations is just the leading order terms in the system-size expansion.

The next-to-leading order gives rise to a Fokker-Planck equation for the fluctuation variables x_1, x_2, x_3

$$\frac{\partial\Pi}{\partial t}=-\sum_{k,l=1}^{3}A_{kl}\frac{\partial(x_l\Pi)}{\partial x_k}+\frac{1}{2}\sum_{k,l=1}^{3}B_{kl}\frac{\partial^2\Pi}{\partial x_k\partial x_l}. \qquad \text{(A11)}$$

Since we are interested in fluctuations about the fixed point $E^*=(\phi_1^*,\phi_2^*,\psi^*)$ defined in Eq. (6) of the determinist model, both matrix $\mathcal{A}=(A_{kl})_{3\times3}$ and $\mathcal{B}=(B_{kl})_{3\times3}$ are evaluated at this fixed point, whose explicit forms are found to be

$$\mathcal{A}=(A_{kl})_{3\times3}=\begin{pmatrix}\frac{\partial f_1}{\partial\phi_1} & \frac{\partial f_1}{\partial\phi_2} & \sqrt{\kappa}\frac{\partial f_1}{\partial\psi}\\ \frac{\partial f_2}{\partial\phi_1} & \frac{\partial f_2}{\partial\phi_2} & \sqrt{\kappa}\frac{\partial f_2}{\partial\psi}\\ 0 & \frac{1}{\sqrt{\kappa}}\frac{\partial f_3}{\partial\phi_2} & \frac{\partial f_3}{\partial\psi}\end{pmatrix}_{(\phi_1=\phi_1^*,\phi_2=\phi_2^*,\psi=\psi^*)}, \qquad \text{(A12)}$$

and

$$\mathcal{B} = (B_{kl})_{3\times 3} = \left(\begin{array}{ccc} B_{11} & B_{12} & 0 \\ B_{21} & B_{22} & 0 \\ 0 & 0 & B_{33} \end{array} \right)_{(\phi_1=\phi_1^*,\phi_2=\phi_2^*,\psi=\psi^*)}, \tag{A13}$$

with

$$\begin{aligned} B_{11} &= \beta\phi_1\phi_2 + \rho\phi_1\psi + \mu(1-\phi_1), \\ B_{22} &= \beta\phi_1\phi_2 + \rho\phi_1\psi + (\mu+\gamma)\phi_2, \\ B_{33} &= \delta\psi + \kappa\tau\phi_2 + \eta\psi, \\ B_{12} &= B_{21} = -\beta\phi_1\phi_2 - \rho\phi_1\psi - \mu\phi_2. \end{aligned}$$

A1.3 Power spectral calculation and its peak

To calculate the power spectra of the fluctuations around the stationary state, we have to make a Fourier analysis, so it is first essential to formulate a set of Langevin equations of the stochastic variables $x_k(t), (k = 1, 2, 3)$. The Langevin equations corresponding to Eq. (A11) are

$$\frac{dx_k}{dt} = \sum_{l=1}^{3} A_{kl}x_l + \xi_k(t), \quad (k, l = 1, 2, 3) \tag{A14}$$

which are three differential equations describing the stochastic behavior of the model at large, but finite N. The variables $x_l(l = 1, 2, 3)$ are stochastic corrections to the deterministic variables s, i and v, and $\xi_k(t)(k = 1, 2, 3)$ are Gaussian white noises with zero mean and a correlation function given by

$$\langle \xi_k(t)\xi_l(t')\rangle = B_{kl}\boldsymbol{\delta}(t - t').$$

Taking the temporal Fourier transform of (A14) gives

$$-\,\mathrm{i}\omega\tilde{x}_k(\omega) = \sum_{l=1}^{3} A_{kl}\tilde{x}_l(\omega) + \tilde{\xi}_k(\omega), \quad (k, l = 1, 2, 3) \tag{A15}$$

with

$$\langle \tilde{\xi}_k(\omega)\tilde{\xi}_l(\omega')\rangle = B_{kl}(2\pi)\boldsymbol{\delta}(\omega + \omega').$$

Actually, this Fourier transform is a system with three coupled linear algebraic equations which can be used to obtain a closed form expression for the power spectra. Therefore, solving Eq. (A15), we obtain

$$\begin{aligned} \tilde{x}_1(\omega) &= \frac{\Lambda_{11}\tilde{\xi}_1 + \Lambda_{12}\tilde{\xi}_2 + \Lambda_{13}\tilde{\xi}_3 - (i\omega)^2\tilde{\xi}_1 + (i\omega)\Theta_1}{\mathcal{D}}, \\ \tilde{x}_2(\omega) &= \frac{\Lambda_{21}\tilde{\xi}_1 + \Lambda_{22}\tilde{\xi}_2 + \Lambda_{23}\tilde{\xi}_3 - (i\omega)^2\tilde{\xi}_2 + (i\omega)\Theta_2}{\mathcal{D}}, \\ \tilde{x}_3(\omega) &= \frac{\Lambda_{31}\tilde{\xi}_1 + \Lambda_{32}\tilde{\xi}_2 + \Lambda_{33}\tilde{\xi}_3 - (i\omega)^2\tilde{\xi}_3 + (i\omega)\Theta_3}{\mathcal{D}}, \end{aligned} \tag{A16}$$

where

$$
\begin{aligned}
\Lambda_{11} &= A_{32}A_{23} - A_{33}A_{22}, \quad \Lambda_{12} = A_{12}A_{33} - A_{13}A_{32}, \\
\Lambda_{13} &= A_{13}A_{22} - A_{12}A_{23}, \quad \Theta_1 = -(A_{22} + A_{33})\tilde{\xi}_l + A_{12}\tilde{\xi}_2 + A_{13}\tilde{\xi}_3; \\
\Lambda_{21} &= A_{21}A_{33}, \quad \Lambda_{22} = -A_{11}A_{33}, \\
\Lambda_{23} &= A_{23}A_{11} - A_{13}A_{21}, \quad \Theta_2 = A_{21}\tilde{\xi}_1 - (A_{11} + A_{33})\tilde{\xi}_2 + A_{23}\tilde{\xi}_3; \\
\Lambda_{31} &= -A_{21}A_{32}, \quad \Lambda_{32} = A_{11}A_{32}, \\
\Lambda_{33} &= A_{12}A_{21} - A_{11}A_{22}, \quad \Theta_3 = A_{32}\tilde{\xi}_2 - (A_{11} + A_{22})\tilde{\xi}_3,
\end{aligned}
$$

and the denominator $\mathcal{D}$ is given by

$$
\begin{aligned}
\mathcal{D}(\omega) &= (\mathrm{i}\omega)^3 + \mathrm{tr}\mathcal{A}(\mathrm{i}\omega)^2 + \Omega(\mathrm{i}\omega) + \det\mathcal{A}, \qquad \text{(A17)} \\
\mathrm{tr}\mathcal{A} &= A_{11} + A_{22} + A_{33}, \\
\Omega &= A_{11}A_{22} + A_{11}A_{33} + A_{33}A_{22} - A_{32}A_{23} - A_{12}A_{21}, \\
\det\mathcal{A} &= A_{11}A_{33}A_{22} - A_{12}A_{21}A_{33} + A_{13}A_{21}A_{32} - A_{11}A_{32}A_{23}.
\end{aligned}
$$

Averaging the squared moduli of $\tilde{x}_k, (k = 1, 2, 3)$ gives the power-spectra of variables S, I and V:

$$
\begin{aligned}
P_S(\omega) = \langle|\tilde{x}_1(\omega)|^2\rangle &= \frac{\alpha_S + B_{11}\omega^4 + \Gamma_1\omega^2}{|\mathcal{D}(\omega)|^2}, \\
P_I(\omega) = \langle|\tilde{x}_2(\omega)|^2\rangle &= \frac{\alpha_I + B_{22}\omega^4 + \Gamma_2\omega^2}{|\mathcal{D}(\omega)|^2}, \\
P_V(\omega) = \langle|\tilde{x}_3(\omega)|^2\rangle &= \frac{\alpha_V + B_{33}\omega^4 + \Gamma_3\omega^2}{|\mathcal{D}(\omega)|^2}, \qquad \text{(A18)}
\end{aligned}
$$

where

$$
\begin{aligned}
|\mathcal{D}(\omega)|^2 &= (\omega^3 - \Omega\omega)^2 + (\mathrm{tr}\mathcal{A}\omega^2 - \det\mathcal{A})^2, \\
\alpha_S &= B_{11}\Lambda_{11}^2 + B_{22}\Lambda_{12}^2 + B_{33}\Lambda_{13}^2 + 2B_{12}\Lambda_{11}\Lambda_{12}, \\
\Gamma_1 &= B_{11}(A_{22}^2 + A_{33}^2) + B_{22}A_{12}^2 + B_{33}A_{13}^2 + 2[B_{11}A_{22}A_{33} - B_{12}A_{12}A_{22} \\
&\quad - B_{12}A_{12}A_{33} + B_{11}\Lambda_{11} + B_{12}\Lambda_{12}]; \\
\alpha_I &= B_{11}\Lambda_{21}^2 + B_{22}\Lambda_{22}^2 + B_{33}\Lambda_{23}^2 + 2B_{12}\Lambda_{21}\Lambda_{22}, \\
\Gamma_2 &= B_{22}(A_{11}^2 + A_{33}^2) + B_{11}A_{21}^2 + B_{33}A_{23}^2 + 2[B_{22}A_{11}A_{33} - B_{12}A_{11}A_{21} \\
&\quad - B_{12}A_{21}A_{33} + B_{12}\Lambda_{21} + B_{22}\Lambda_{22}]; \\
\alpha_V &= B_{11}\Lambda_{31}^2 + B_{22}\Lambda_{32}^2 + B_{33}\Lambda_{33}^2 + 2B_{12}\Lambda_{31}\Lambda_{32}, \\
\Gamma_3 &= B_{33}(A_{11}^2 + A_{22}^2) + B_{22}A_{32}^2 + 2[B_{33}A_{11}A_{22} + B_{33}\Lambda_{33}].
\end{aligned}
$$

By using these methods, we can analytically predict the epidemic outbreaks and fade-outs on a certain disease, as done by Alonso *etal* [21] for several childhood diseases.

A2 Stability analysis of fixed points E^0 and E^* of system (A6)

First, we give the stability analysis on equilibria E^0, the Jacobian matrix of which given by

$$J_1 = \begin{pmatrix} -\mu & -\beta & -\rho \\ 0 & \beta-\mu-\gamma & \rho \\ 0 & \kappa\tau & \delta-\eta \end{pmatrix} \quad ,$$

and its characteristic polynomial can be written as

$$(\lambda+\mu)\{\lambda^2 + [(\eta-\delta)+(\gamma+\mu-\beta)]\lambda + (\eta-\delta)(\gamma+\mu-\beta) - \kappa\rho\tau\}.$$

Hence, it is easy know that the trivial fixed point E^0 is stable if and only if the conditions $(\eta-\delta)+(\gamma+\mu-\beta) > 0$ and $(\eta-\delta)(\gamma+\mu-\beta) - \kappa\rho\tau > 0$ are satisfied, which equal to the condition of basic reproduction number, i.e., $\mathcal{R}_0 < 1$, and $\mathcal{R}_0 = \frac{\beta}{\mu+\gamma} + \frac{\kappa\rho\tau}{(\eta-\delta)(\mu+\gamma)}$.

Second, the stability analysis on equilibria E^*. The Jacobian matrix at E^* is

$$J_2 = \begin{pmatrix} -\beta\phi_2^* - \rho\psi^* - \mu & -\beta\phi_1^* & -\rho\phi_1^* \\ \beta\phi_2^* + \rho\psi^* & \beta\phi_1^* - \mu - \gamma & \rho\phi_1^* \\ 0 & \kappa\tau & \delta-\eta \end{pmatrix} \quad ,$$

where where the stars indicate the values at equilibrium E^*, which are $\phi_1^* = \frac{1}{\mathcal{R}_0}, \phi_2^* = \frac{\mu}{\mu+\gamma}(1 - \frac{1}{\mathcal{R}_0})$, and $\psi^* = \frac{\kappa\mu\tau}{(\eta-\delta)(\mu+\gamma)}(1-\frac{1}{\mathcal{R}_0})$.

The characteristic polynomial of Jacobian matrix J_2 is written as a cubic polynomial about λ denoted as $L(\lambda)$:

$$L(\lambda) = \lambda^3 + a\lambda^2 + b\lambda + c,$$

all of the coefficients of which are

$$\begin{aligned} a &= -\frac{\beta}{\mathcal{R}_0} + \gamma(-\delta+\eta) + 2\mu + \{\frac{\beta\mu}{\gamma+\mu} - \frac{\beta\mu}{\mathcal{R}_0(\gamma+\mu)}\} + \{\frac{\kappa\mu\rho\tau}{(-\delta+\eta)(\gamma+\mu)} - \frac{\kappa\mu\rho\tau}{\mathcal{R}_0(-\delta+\eta)(\gamma+\mu)}\} \\ &= (\eta-\delta) + \mu\mathcal{R}_0 + \gamma + \mu - \frac{\beta}{\mathcal{R}_0}, \\ &> (\eta-\delta) + \mu\mathcal{R}_0 > 0, \end{aligned} \tag{A19}$$

$$
\begin{aligned}
b &= (\frac{\beta\delta}{\mathcal{R}_0} - \frac{\beta\eta}{\mathcal{R}_0} - \frac{\kappa\rho\tau}{\mathcal{R}_0}) - \frac{\beta\mu}{\mathcal{R}_0} + (\gamma\eta - \gamma\delta) + (\mu^2 + \gamma\mu) + (2\eta\mu - 2\delta\mu) \\
&\quad + \{\frac{\beta\gamma\mu}{\gamma+\mu} - \frac{\beta\gamma\mu}{\mathcal{R}_0(\gamma+\mu)}\} + \{-\frac{\beta\delta\mu}{\gamma+\mu} + \frac{\beta\delta\mu}{\mathcal{R}_0(\gamma+\mu)} + \frac{\beta\eta\mu}{\gamma+\mu} - \frac{\beta\eta\mu}{\mathcal{R}_0(\gamma+\mu)}\} \\
&\quad + \{\frac{\beta\mu^2}{\gamma+\mu} - \frac{\beta\mu^2}{\mathcal{R}_0(\gamma+\mu)}\} + \{\frac{\kappa\gamma\mu\rho\tau}{(-\delta+\eta)(\gamma+\mu)} - \frac{\kappa\gamma\mu\rho\tau}{\mathcal{R}_0(-\delta+\eta)(\gamma+\mu)}\} \\
&\quad + \{-\frac{\kappa\delta\mu\rho\tau}{(-\delta+\eta)(\gamma+\mu)} + \frac{\kappa\delta\mu\rho\tau}{\mathcal{R}_0(-\delta+\eta)(\gamma+\mu)} + \frac{\kappa\eta\mu\rho\tau}{(-\delta+\eta)(\gamma+\mu)} - \frac{\kappa\eta\mu\rho\tau}{\mathcal{R}_0(-\delta+\eta)(\gamma+\mu)}\} \\
&\quad + \{\frac{\kappa\mu^2\rho\tau}{(-\delta+\eta)(\gamma+\mu)} - \frac{\kappa\mu^2\rho\tau}{\mathcal{R}_0(-\delta+\eta)(\gamma+\mu)}\} \\
&= \mu\{(\eta-\delta) + (\gamma+\mu)\}\mathcal{R}_0 - \mu\frac{\beta}{\mathcal{R}_0}, \\
&> \mu(\eta-\delta)\mathcal{R}_0 + \mu(\gamma+\mu)(\mathcal{R}_0 - 1) > 0, \qquad \text{(A20)}
\end{aligned}
$$

both of the above inequations (A19) and (A20) are obtained in the condition:
$\mathcal{R}_0 = \frac{\beta}{\mu+\gamma} + \frac{\kappa\rho\tau}{(\eta-\delta)(\mu+\gamma)} \Rightarrow \mathcal{R}_0 > \frac{\beta}{\gamma+\mu} \Rightarrow -\frac{\beta}{\mathcal{R}_0} > -(\gamma+\mu)$.

$$
\begin{aligned}
c &= (\frac{\beta\delta\mu}{\mathcal{R}_0} - \frac{\beta\eta\mu}{\mathcal{R}_0} - \frac{\kappa\mu\rho\tau}{\mathcal{R}_0}) + \{(\gamma\eta\mu - \gamma\delta\mu) + (\eta\mu^2 - \delta\mu^2)\} \\
&\quad + \{-\frac{\beta\gamma\delta\mu}{\gamma+\mu} + \frac{\beta\gamma\delta\mu}{\mathcal{R}_0(\gamma+\mu)} + \frac{\beta\gamma\eta\mu}{\gamma+\mu} - \frac{\beta\gamma\eta\mu}{\mathcal{R}_0(\gamma+\mu)}\} + \{-\frac{\beta\delta\mu^2}{\gamma+\mu} + \frac{\beta\delta\mu^2}{\mathcal{R}_0(\gamma+\mu)} + \frac{\beta\eta\mu^2}{\gamma+\mu} - \frac{\beta\eta\mu^2}{\mathcal{R}_0(\gamma+\mu)}\} \\
&\quad + \{-\frac{\kappa\gamma\delta\mu\rho\tau}{(-\delta+\eta)(\gamma+\mu)} + \frac{\kappa\gamma\delta\mu\rho\tau}{\mathcal{R}_0(-\delta+\eta)(\gamma+\mu)} + \frac{\kappa\gamma\eta\mu\rho\tau}{(-\delta+\eta)(\gamma+\mu)} - \frac{\kappa\gamma\eta\mu\rho\tau}{\mathcal{R}_0(-\delta+\eta)(\gamma+\mu)}\} \\
&\quad + \{-\frac{\kappa\delta\mu^2\rho\tau}{(-\delta+\eta)(\gamma+\mu)} + \frac{\kappa\delta\mu^2\rho\tau}{\mathcal{R}_0(-\delta+\eta)(\gamma+\mu)} + \frac{\kappa\eta\mu^2\rho\tau}{(-\delta+\eta)(\gamma+\mu)} - \frac{\kappa\eta\mu^2\rho\tau}{\mathcal{R}_0(-\delta+\eta)(\gamma+\mu)}\} \\
&= \mu(\eta-\delta)(\gamma+\mu)(\mathcal{R}_0 - 1) > 0,
\end{aligned}
$$

Then we can obtain

$$
\begin{aligned}
ab - c &> \{(\eta-\delta) + \mu\mathcal{R}_0\}\{\mu(\eta-\delta)\mathcal{R}_0 + \mu(\gamma+\mu)(\mathcal{R}_0 - 1)\} - \{\mu(\eta-\delta)(\gamma+\mu)(\mathcal{R}_0 - 1)\} \\
&= \mu(\eta-\delta)^2\mathcal{R}_0 + (\eta-\delta)(\mu\mathcal{R}_0)^2 + \mu^2(\gamma+\mu)\mathcal{R}_0(\mathcal{R}_0 - 1) > 0.
\end{aligned}
$$

According to the criterion of Routh-Hurwitz, the conditions $a > 0$, $ab - c > 0$, and $c > 0$ are satisfied, thus the equilibrium E^* is stable.

A3 Environmental transmission

The environmental transmission rate is defined as the per capita rate at which susceptible individuals get infected through the environmental transmission route. This rate will be higher the higher the concentration of virion particles in the environment. This is so because individual birds are continuously exposed to the disease via ingestion of lake water while feeding. In general,

whenever dose-response curves have been empirically measured, it has been observed that the probability of infection indeed depends on the "dose", or quantity of infection agents ingested by individuals. From these curves, a ID_{50} is defined as the virion concentration at which half of the tested population gets infected.

In order to approximate how the environmental transmission rate depends on virion concentration in the water we make the following argument.

If N birds are exposed to contaminated lake water with a virion concentration V in the water, and monitored for a time t, the number, n, of birds that will develop the infection will be distributed following a binomial distribution:

$$P(n) = \binom{N}{n} p^n \, (1-p)^{N-n} \tag{A21}$$

where the probability p can be written as:

$$p = \alpha \, V \, t \tag{A22}$$

and α is the volume of water ingested per unit time per individual bird. Notice that p is a number (total ingested virion particles), lacking physical dimensions, as corresponds to a probability. However, this model only works for times short enough to make $p < 1$.

The limit of large bird populations (N), and low total virion ingestion ($\alpha V t$), leads to the familiar Poison distribution, under which the probability of observing n individual infections during a finite time t is:

$$P(n) = \frac{(\alpha \, V \, t)^n}{n!} \exp(-\alpha \, V \, t). \tag{A23}$$

Notice that the probability of observing at least one infection in a time interval t is $1 - P(0)$. This can be seen as a the pre-factor reducing a faster asymptotic per capita rate, ρ_a, at which susceptible individuals would get infected through the environmental transmission route if the virion concentration in the water were very big, effectively infinite. So, we can write:

$$\hat{\rho}(V) = \rho_a(1 - \exp(-\alpha_t \, V)) \tag{A24}$$

where we have defined $\alpha_t \equiv \alpha \, t$ to make contact with the expression used by Breban et al's (2009).

Also $(1 - \exp(-\alpha \, V \, t))$ can be regarded at the probability distribution function describing the time until an individual gets infected when ingesting water at rate α, contaminated with viruses at a concentration V. Therefore, this waiting time is, in fact, a random variable governed by the exponential distribution. As a consequence, the instantaneous ingestion rate α can be related to the ID_{50}, as defined above:

$$\alpha = \frac{\log_e(2)/ID_{50}}{T} \tag{A25}$$

where ID_{50} is virion concentration at 50% of infection and the time T is the exposure time dose-response tests take. This equation allows to approximate α through typical dose-response

experiments. By exposing individuals to higher and higher viral concentrations, the same dose-response experiments can also inform about the value of the asymptotic value ρ_a.

Furthermore, Eq. (A24) can be approximated by:

$$\hat{\rho}(V) = \rho_a \, \alpha \, V \, t \tag{A26}$$

as long as $\alpha \, V \, t$ is small, in accordance with our initial assumption. In addition, what is clear from Eq. (A26) is that, within the range of this approximation, the environmental transmission rate, $\hat{\rho}(V)$, can be assumed to be proportional to the concentration of virion particles in the the water, V. In fact, throughout our paper, we have used this proportionality, expressed as:

$$\hat{\rho}(V) \approx \frac{V}{N_V} \tag{A27}$$

where N_V is a scaling factor for the virion concentration in the environment. As you see from Eq. (A26), the proportionality factor is, in the context of our approximation, the product $(\alpha \, t \, \rho_a)$.

The proportionality factor in Eq. (A27) is actually one of our model parameters, ρ (see our Eq (5) in the main text). The following argument and data from the literature allow us to obtain an estimation of some of our model parameters.

The volume of water ingested per unit time per individual bird has been estimated to be of about 2500 to 25000 liters/year (Bennett and Hughes, 2003). Experimental studies show that ID_{50} take values of $10^{1.8}$ to $10^{5.7}$ viral particles per ml (Lu et al 2004 and Ito et al 1995). With this data in mind, and using the relation between α and ID_{50} above, we can write:

$$\rho = \alpha \, t \, \rho_a = \frac{\log_e(2)}{ID_{50}} \frac{t}{T} \rho_a \tag{A28}$$

On one hand, we can identify $N_V \approx ID_{50}$, and, on the other, t and T are both short times that can be considered of the same order, $t/T \approx 1$. So we can finally write the environmental transmission rate as:

$$\hat{\rho}(V) = \rho_a \log_e(2) \frac{V}{N_V} \tag{A29}$$

This shows that the model parameter ρ corresponds, in the context of our approximation, to the asymptotic value ρ_a times $\log_e(2)$. Moreover, this argument supports our choice of roughly $N_V = 10^5$ throughout our paper (see Table 2), and points to ways of estimating our model parameter ρ by measuring the asymptotic rate at which individuals get infected when they are exposed at higher and higher concentrations of virion particles in the water they drink. In fact, in this context (see Eq. (A29)), the parameter ρ represents approximately the rate at which individual ducks get sick at viral concentrations in the water about the ID_{50}. Given the quantity of water birds drink daily (7 to 70 l per day), at moderate concentrations of viral particles in the water, they ingest during a day several times the dose that would infect them if they took that dose as a shot in one go. However, probably the same quantity of viral particles have a very mild effect if they are diluted during a day compared to concentrated in a single shot. Experimental tests to estimate ID_{50} are usually done by providing a single shot of highly concentrated infected water. This makes it difficult to translate lab results to the field.

Finally, notice that the exponentially saturating functional form for the environmental transmission (see Eq. A24), suggested by Breban et al (2009), relies, as these authors already noted, on the Markovian assumption that the probability that a duck escapes infection when exposed to a virial concentration V does not depend on how much that duck has been exposed to the disease in the past, so it can be modeled by the exponential distribution. However, the derivation presented here makes clear the different approximations that such a representation involves. In addition, this simple analysis has also allowed us to give a rough estimate for the typical values of some of our model parameters.

A4 Sensitivity analysis

To better understand the effects of the uncertainty of parameter values on fluctuating periodicity, we performed a sensitivity analysis of the periodicity with respect to two model parameters: the environmental transmission rate ρ, and the host recovery rate, γ. In Fig. 1, we show the variation of the dominant period for different values of ρ and γ. The dominant period was calculated by averaging wavelet power spectra (see section wavelet power spectrum in the main text) over 20 independent simulations. On the left panel, we can see that decreased environmental transmission rate can significantly lengthen the period of the disease outbreak ($P < 0.001$). By comparison, ρ should increase to higher values to see a significant decrease in the main periodicity of time-series. Since both rates are given in the same units, we can also safely say that periodicity is less sensitive to changes in the the recovery rate γ than it is to changes in the environmental transmission rate ρ.

We also checked that when the parameter δ, representing the reproduction of the infectious agents in alternative hosts different from the focal host, tend to zero, our main conclusions hold. For instance, when we plot the theoretical PSDs given by Eqs (7) by taking $\delta = 0$, the reported shift to longer dominant periods as environmental transmission decreases is also seen (comparare Fig. 2 with Fig. 3 in the main text).

References

Breban R., Drake J. M., Stallknecht D. E., Rohani P. (2009) The Role of Environmental Transmission in Recurrent Avian Influenza Epidemics. PLoS Comput. Biol. 5(4): e1000346.

Bennett D. C., Hughes M. R. (2003) Comparison of renal and salt gland function in three species of wild ducks. J. Exp. Biol. 206: 3273–3284.

Lu, H., Castro, A. E. (2004) Evaluation of the infectivity, length of infection, and immune response of a low-pathogenic H7N2 avian influenza virus in specific pathogen-free chickens. Avian Dis. 48: 263–270.

Ito, T., Okazaki, K., et al. (1995) Perpetuation of influenza A viruses in Alaskan waterfowl reservoirs. Arch. Virol. 140: 1163–1172.

Hurwitz, A. (1895). On the conditions under which an equation has only roots with negative real parts. Mathematische Annalen, vol. 46, pp. 273–284.

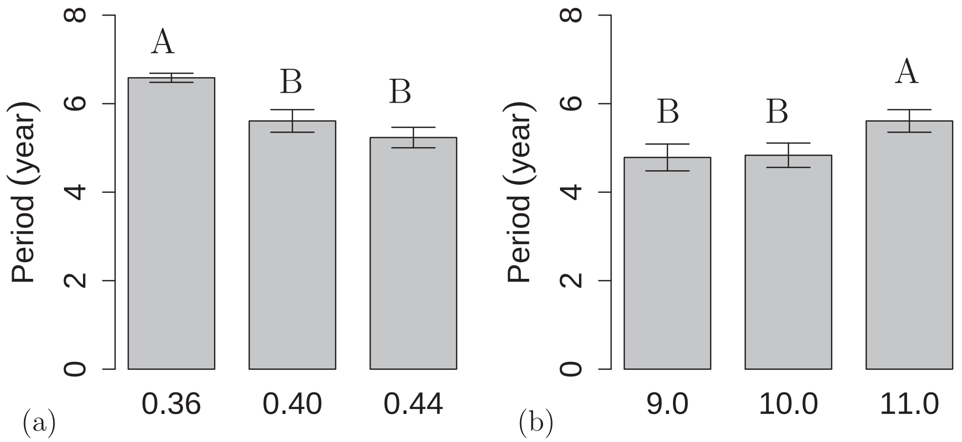

Figure S1. (Online version in colour) Sensitivity analysis on the influence of parameters ρ (a) and γ (b) on the periodicity of disease outbreaks. We analyze the effect of $\pm10\%$ changes in the parameter values on the dominant period of the infectious time series. We took as reference values $\rho = 0.4$ and $\gamma = 10$, represented on the central bars in panel (a) and (b), respectively. Other parameter values are listed in Table 2. Error bars denote ±1 mean standard errors. The capital letters A and B denote whether or not there are significant differences in the average dominant period across the different *insilico* experiments based on Tukey's honest significant difference.

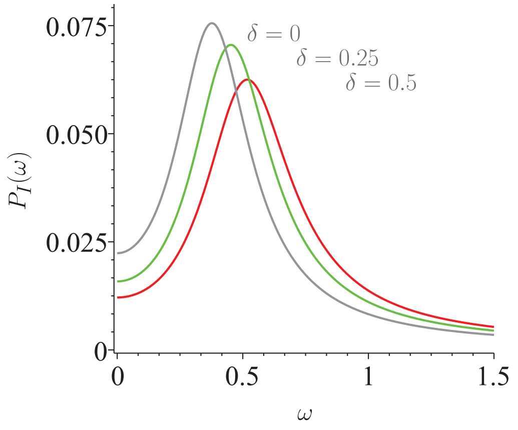

Figure S2. (Online version in colour) The qualitative behavior of the PSD when the parameter δ tends to zero is the same as reported in the main text. The frequency values at which these curves peak do not differ significantly from those reported in Fig. 3b
